# Powered Exoskeleton Gait Training and Hip Rate of Force Development in Chronic Hypoxic-Ischemic Encephalopathy: A Case Study

**DOI:** 10.3390/brainsci16070688

**Published:** 2026-06-30

**Authors:** Yukyoung Won, Junggi Hong

**Affiliations:** 1 Major in Sports Medicine, Department of Medicine, CHA University of Medicine and Science, Pocheon 11160, Republic of Korea; itsumo00@naver.com; 2 Graduate School of Sports Medicine, CHA University of Medicine and Science, Pocheon 11160, Republic of Korea

**Keywords:** hypoxic-ischemic encephalopathy, powered exoskeleton, rate of force development, sit-to-stand, hip neuromuscular strength, case report, neurorehabilitation

## Abstract

**Highlights:**

**What are the main findings?**

- Six weeks of powered exoskeleton gait training selectively enhanced the hip extension rate of force development (RFD) by up to +188%, exceeding the relative gains observed in maximal muscle strength.
- Body mass-normalized RFD during sit-to-stand increased by 250%, representing the largest relative improvement across all outcome measures.

**What are the implications of the main findings?**

- Powered exoskeleton gait training may contribute to adaptive changes in hip-centered neuromuscular output strategies, improving time-dependent force generation capacity beyond the effects of repetitive ambulation practice alone.
- Rate of force development (RFD) should be incorporated as a key assessment and intervention target in the rehabilitation of patients with chronic central nervous system injury.

**Abstract:**

Background: Evidence on powered wearable exoskeleton gait training in patients with chronic hypoxic-ischemic encephalopathy (HIE) is virtually absent, and existing studies have focused on macroscopic functional outcomes while neglecting joint-level neuromuscular force-generation characteristics such as rate of force development (RFD). Objective: To examine the effects of a six-week powered exoskeleton gait training program on isometric hip strength and RFD, sit-to-stand (STS) performance, frontal-plane hip strength, and center-of-pressure (CoP) dynamics in a patient with chronic HIE-induced quadriparesis. Methods: A case report with pre- and post-intervention evaluation was conducted. A 47-year-old male with chronic HIE-induced quadriparesis (onset 2017) completed 18 sessions (three per week, six weeks) of powered lower-limb exoskeleton gait training. Outcomes included isometric hip peak force and RFD (DynaMo, Vald Performance), STS peak force and body mass-normalized RFD (ForceDecks, Vald Performance), frontal-plane hip strength (ForceFrame, Vald Performance), and CoP path length and mean velocity. Results: Hip extension peak force increased by 247–256% bilaterally, and hip extension RFD increased by 174–188%, whereas hip flexion peak force showed minimal change (+3.3–5.2%). Body mass-normalized STS RFD increased by 250% (10 to 35 N·s^−1^·kg^−1^), representing the largest relative gain. Hip abduction strength increased by 27.1–36.8% with improved bilateral symmetry; hip adduction imbalance reversed from right to left dominance. CoP path length and mean velocity each decreased by 3.7%. Conclusions: Six weeks of powered exoskeleton gait training selectively enhanced time-dependent neuromuscular output—particularly RFD—beyond maximal strength gains, with meaningful improvements in functional weight acceptance during STS. These findings support exoskeleton-based training as a promising rehabilitation strategy for patients with chronic CNS injury.

## 1. Introduction

Hypoxic-ischemic encephalopathy (HIE) results from global cerebral hypoxia that disrupts cellular metabolic homeostasis, producing necrosis at the lesion core and ischemic damage in the surrounding penumbra [1]. Because this injury affects descending motor pathways including the corticospinal tract, the resulting impairment reflects deficits in sensory integration and neuromuscular control, not merely reduced muscle strength [2,3].

Patients with HIE-related tetraparesis exhibit proprioceptive sensory deficits, spasticity, hyperreflexia, delayed motor unit recruitment, and asymmetric muscle activation [3,4]. The central deficit is therefore not simply reduced maximal force but a qualitative decline in neuromuscular output—specifically, reduced rate of force development (RFD) and delayed force onset [5]. RFD, the capacity to generate force within the initial 50–100 ms, is a key determinant of successful weight acceptance during early stance and sit-to-stand (STS) [5,6], and reduced RFD is associated with decreased mobility and increased fall risk after brain injury [7].

Gait stability also depends on frontal-plane hip abductor function, which maintains pelvic levelness and limits lateral trunk displacement during single-limb support; impairment leads to compensatory trunk strategies and gait asymmetry [8,9]. STS performance, central to functional independence, is similarly influenced by lower-extremity strength, weight distribution, and task-related factors such as seat height and armrest use [10], and patients with CNS injury show reduced, asymmetric weight-bearing on the paretic limb during STS [11].

Quiet standing reflects sensory integration and postural control strategies. Center-of-pressure (CoP) dynamics are used to quantify postural control after neurological injury [12]; reduced CoP displacement reflects a stiffening strategy, whereas excessive variability reflects an unstable strategy [12,13].

Reduced ambulatory capacity drives a sedentary lifestyle and secondary complications, including decreased cardiopulmonary function and bone mineral density [14]. Restoring gait is therefore a primary rehabilitation goal, but conventional approaches—task-oriented and body weight-supported treadmill training—are limited by repetition intensity and therapist dependency [15], and stationary robotic exoskeletons remain constrained by pre-programmed trajectories [16].

Powered wearable exoskeletons have emerged as an alternative, enabling repetitive, symmetrical gait practice in real-world environments [17]. Systematic reviews indicate that exoskeleton-based training can improve gait velocity, mobility, and functional independence [17,18], with recent studies also reporting changes in muscle activation patterns and weight-bearing symmetry [18,19].

According to motor control theory, efficient movement depends less on absolute muscle force than on the temporal organization of muscle activation and sensory reweighting strategies [20], capacities that are disrupted following CNS injury [3,12]. Rehabilitation should therefore target the temporal characteristics of neuromuscular output, not strength alone.

However, existing exoskeleton literature has focused predominantly on macroscopic outcomes such as gait speed, and studies comprehensively examining joint-level RFD, frontal-plane hip stability, STS weight acceptance, and CoP dynamics remain limited.

Therefore, this case report examined the effects of a six-week powered wearable exoskeleton gait training intervention in a patient with chronic HIE-induced quadriparesis on: (1) joint-level neuromuscular force-generation characteristics (RFD); (2) frontal-plane hip stability; (3) functional weight acceptance capacity during sit-to-stand; and (4) postural control during quiet standing.

## 2. Materials and Methods

### 2.1. Study Design

This study was conducted as a case report with pre- and post-intervention evaluation in one patient with chronic HIE-induced quadriparesis. A six-week powered wearable exoskeleton gait training intervention was administered, and the same assessment protocol was applied once before and once after the intervention to quantitatively describe changes in isometric muscle strength, functional performance, and static balance. All assessments were conducted under identical measurement conditions by the same examiner. As only a single baseline assessment was performed rather than multiple sequential baseline measurements, this design does not meet the criteria for an experimental single-case (single-subject) design and should be interpreted as a descriptive case report.

### 2.2. Participant

A 47-year-old male (body mass 65 kg, height 172 cm) with diffuse hypoxic brain damage (ICD-10: G93.1) resulting in chronic quadriparesis (ICD-10: G82.5) participated in this study. The hypoxic brain injury occurred in 2017 due to cardiac arrest-induced cerebral hypoxia. The participant had a documented history of extensive hypoxic brain injury involving global cortical and subcortical regions without a discrete focal lesion. The participant was in the chronic phase, having passed more than seven years since symptom onset at the time of the study. Sensory assessment revealed reduced proprioception and light touch sensation in all four extremities, with greater impairment in the lower limbs. The participant was not receiving any concurrent physical or occupational therapy during the six-week intervention period, and no neurological medications were administered throughout the study. The participant had been receiving continuous rehabilitation treatment prior to enrollment but had plateaued in functional progress.

With respect to motor function, the participant demonstrated incomplete voluntary muscle contraction in all four extremities (i.e., quadriparesis rather than complete paralysis), was unable to ambulate independently, but was capable of repetitive gait training with an assistive device. This profile is consistent with partial preservation of the corticospinal tract and residual motor unit recruitment capacity.

Language function assessment revealed an Aphasia Quotient (AQ) of 73.1 (66%) on the Korean version of the Western Aphasia Battery (K-WAB), with a receptive language index of 85.5 and an expressive language index of 83.3. The Korean Boston Naming Test (K-BNT) yielded a raw score of 39 (below the 1st percentile; language quotient 65), indicating moderate anomic aphasia. Articulation assessment revealed consonant accuracy of 86% and vowel accuracy of 80%, representing mild articulatory imprecision.

The participant received a full explanation of the study purpose and procedures and provided voluntary written informed consent.

### 2.3. Intervention

The intervention consisted of repetitive gait training using a powered wearable lower-limb exoskeleton, conducted three sessions per week over six weeks (18 sessions total), with each session lasting approximately 15–25 min (excluding pre-session clinical assessment; see below). All gait training was performed under the supervision of a trained physical therapist.

The powered lower-limb exoskeleton used in this study was the Samsung Bot Fit (Samsung Electronics, Suwon, Republic of Korea). This device was selected for several reasons: at the time of the study, it was the first overground powered wearable lower-limb exoskeleton available in Korea that offered real-time gait data monitoring and session management through a dedicated mobile application, enabling the objective tracking of training parameters and facilitating communication between the therapist and patient. The device was equipped with electric motors at the hip and knee joints and incorporated an inertial measurement unit (IMU) together with gait-phase detection sensors to recognize the user’s gait cycle in real time. It applied an assist-as-needed control algorithm that modulated assistive torque according to the user’s residual motor capacity, providing hip extension and flexion assistance only to the extent necessary when voluntary movement was detected. This approach preserved active muscle engagement while promoting a repetitive and relatively symmetrical gait pattern. The device additionally incorporated a fall-prevention support system and an emergency stop function to ensure participant safety throughout training.

Each session comprised three phases: warm-up, main training, and cool-down. During the warm-up phase (3–5 min), weight-shifting exercises and repetitive STS movements were performed to pre-activate hip extension and facilitate weight acceptance capacity. During the main training phase (10–15 min), repetitive exoskeleton-assisted gait was performed, with the level of assistance adjusted progressively according to the participant’s functional capacity. In the initial phase, emphasis was placed on achieving stable weight acceptance; subsequently, assistive torque was progressively reduced with the goals of improving bilateral symmetry and increasing propulsive force generation. During the cool-down phase (2–5 min), fatigue was assessed and performance feedback was provided.

**Figure 1:**
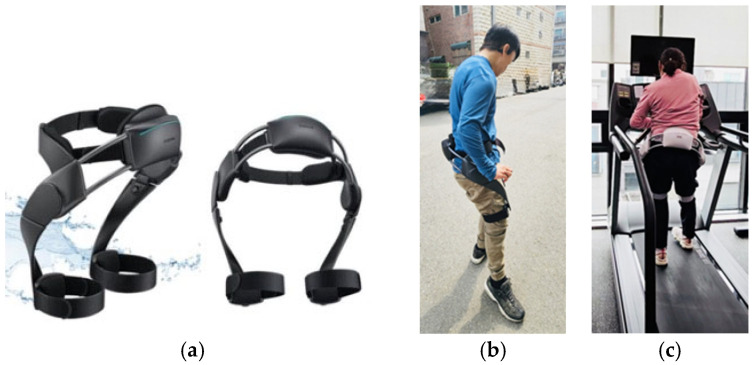
Powered wearable exoskeleton and gait training setup. (**a**) Front and rear views of the Samsung Bot Fit powered lower-limb exoskeleton. (**b**) Lateral view of the participant wearing the exoskeleton during overground gait training. (**c**) Posterior view of a different individual demonstrating treadmill-based use of the exoskeleton, shown for illustrative reference only and not depicting the study participant. Panel (**b**) is a photograph of the actual study participant, taken with written informed consent. Panel (**c**) shows a different individual and is included solely to illustrate device positioning during treadmill-based training.

Prior to each training session, and separate from the 15–25-min training window, the therapist conducted a brief clinical assessment of the participant’s joint range of motion, lower-extremity muscle strength, sensory status, and gait readiness, and verified correct device fitting and alignment. Gait training was performed overground on a level indoor surface under standard conditions; on days when weather precluded outdoor use of the corridor, treadmill-based training was substituted, maintaining the same session structure and duration. During training, safety monitoring was conducted while verbal and tactile cues were provided to facilitate motor learning—specifically weight shifting, heel strike timing, and hip extension initiation. Assistive torque levels were adjusted to maximize voluntary muscle activation in accordance with the participant’s performance. Following each session, changes in gait pattern were documented and goals for the subsequent session were established. The exoskeleton device and the overground and treadmill-based training setups are shown in Figure 1.

### 2.4. Outcome Measures

#### 2.4.1. Isometric Muscle Strength and RFD

**Figure 2:**
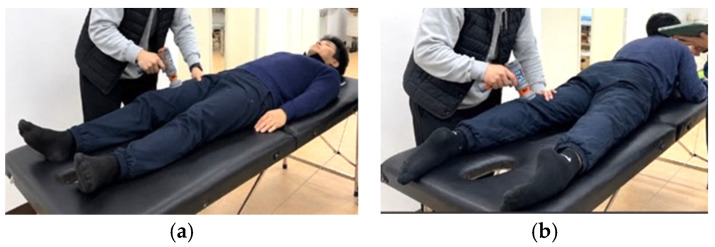
(**a**) Hip flexion strength test: supine position. (**b**) Hip extension strength test: prone position. Isometric hip flexion and extension strength assessment using the handheld DynaMo. Photographs depict the actual study participant, taken with written informed consent.

Isometric muscle strength was assessed using a load-cell-based handheld dynamometer (DynaMo, Vald Performance, Brisbane, Australia). The dynamometer was held manually by the examiner, who applied manual counter-force to resist the participant’s effort (hand-held dynamometer method). Force data were collected through the device’s integrated software (DynaMo app, version 1.9.0) and analyzed based on force–time curves. The testing postures for hip flexion and extension are shown in Figure 2.

The DynaMo was used to assess sagittal-plane hip muscle strength: hip flexion in the supine position with the hip at approximately 45° flexion and the knee extended, and hip extension in the prone position with the knee at approximately 30° flexion (short lever). Although hip abduction was also initially recorded using the DynaMo as a supplementary check, the primary frontal-plane hip abduction and adduction data reported in this study were obtained using the ForceFrame system (see Section 2.4.4), which provides superior positional standardization and mechanical stability for frontal-plane isometric testing. Accordingly, Table 1 reports the DynaMo-derived sagittal-plane data only (hip flexion and extension), whereas the corresponding ForceFrame-derived frontal-plane data (hip abduction and adduction) are presented separately in Section 3.3. Each movement was performed three times, and the maximum value across trials was used for analysis. Rest intervals between trials were self-selected, with the participant indicating readiness before each subsequent attempt to minimize fatigue-related confounding.

The following variables were derived:

- Peak force (N)
- Rate of force development (RFD, N/s)

RFD was calculated as the slope of the force–time curve over the initial 0–200 ms window, interpreted as an index of neuromuscular output characteristics during the early weight acceptance phase.

#### 2.4.2. Sit-to-Stand (STS)

**Figure 3:**
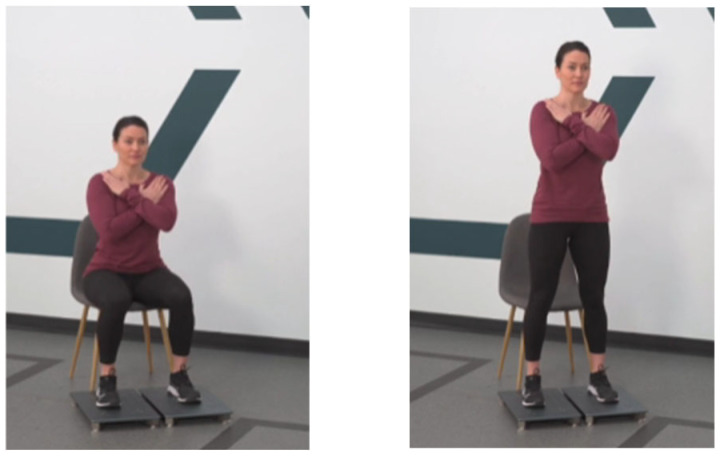
Sit-to-stand performance assessment using ForceDecks. Representative demonstration of the sit-to-stand assessment posture on the dual force plate system, reproduced from the manufacturer’s (Vald Performance) reference materials; images are not photographs of the study participant.

STS performance was assessed using a dual force plate system (ForceDecks, Vald Performance, Brisbane, Australia). The participant stood up from a chair of standardized height with each foot positioned on a separate force plate. Three trials were performed and the maximum value was used for analysis. The assessment posture is illustrated in Figure 3.

The following variables were derived:

- Initial RFD (0–200 ms)
- Peak force (N)
- Body mass–normalized RFD (N·s^−1^·kg^−1^)

#### 2.4.3. Static Balance Assessment

**Figure 4:**
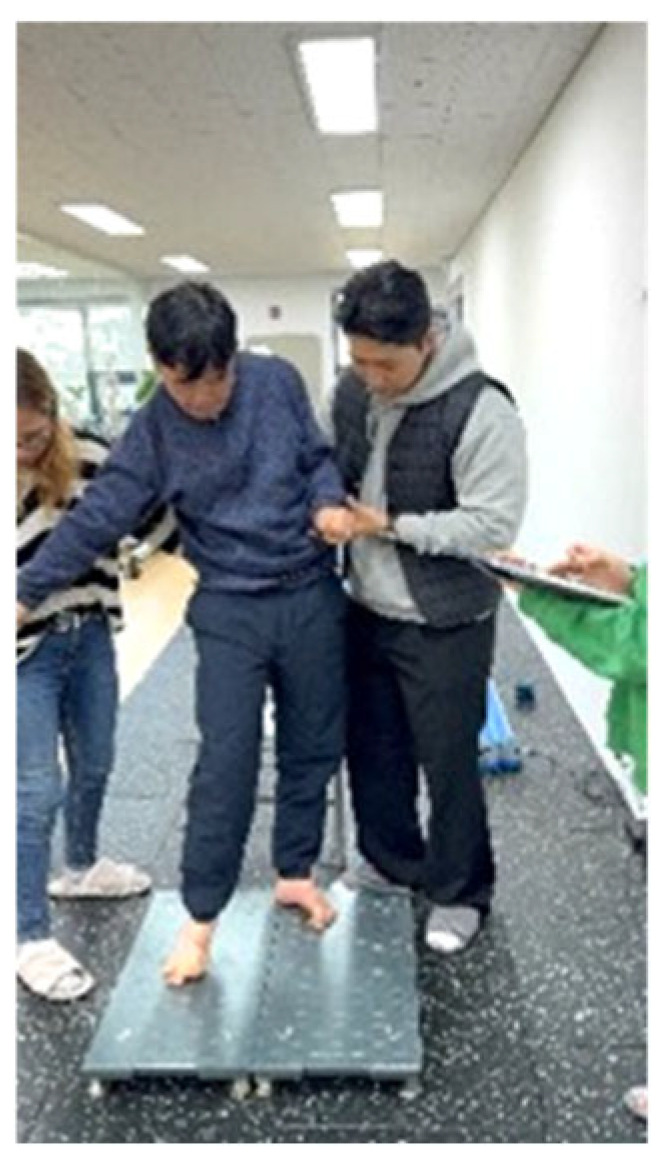
Quiet Stand performance assessment using ForceDecks. Photographs depict the actual study participant, taken with written informed consent.

Static balance was assessed using the same force plate system under eyes-open (EO) and eyes-closed (EC) conditions, each measured for 30 s. The quiet standing assessment setup is shown in Figure 4.

The following variables were derived:

- CoP path length (mm)
- Mean velocity (mm/s)

Static balance was interpreted as a supplementary outcome variable reflecting sensory integration strategies and postural control efficiency.

#### 2.4.4. Frontal-Plane Hip Strength

Frontal-plane hip muscle strength was assessed using an isometric strength testing system (ForceFrame, Vald Performance, Brisbane, Australia). The participant was positioned in a standardized seated posture with the hip and knee joints flexed to approximately 90°. The measurement pad was placed at the distal thigh to assess maximal isometric hip abduction and adduction force bilaterally.

**Figure 5:**
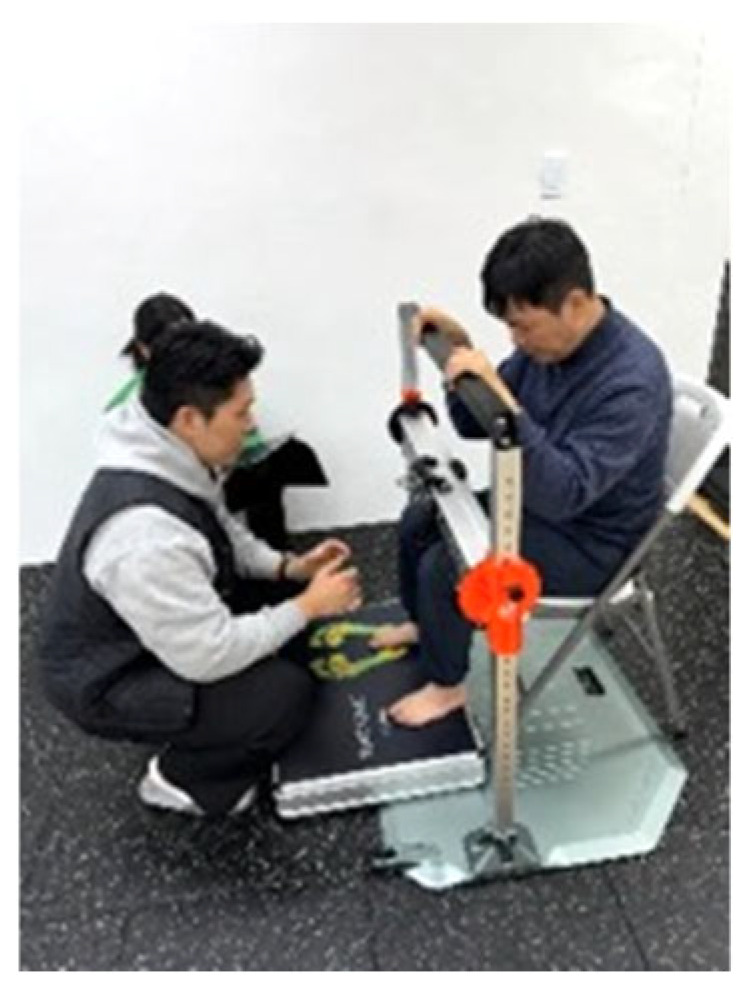
Hip adduction/abduction performance assessment using ForceFrame. Photographs depict the actual study participant, taken with written informed consent.

Each movement was performed three times, and the maximum value across trials was used for analysis; adequate rest was provided between trials. The testing posture is shown in Figure 5.

The following variables were derived:

- Maximum force (N)
- Inter-limb imbalance (%): calculated as ((dominant − non-dominant)/dominant) × 100.

### 2.5. Data Analysis

Given the case report design, the following analyses were performed:

- Percentage change from pre- to post-intervention
- Visual inspection of force–time curves
- Evaluation of clinically meaningful change

Percentage change was calculated as follows: % change = ((post − pre)/pre) × 100. Given the case report design with a single baseline assessment, group-level statistical analyses were not performed; results are presented using descriptive statistics and clinical interpretation.

### 2.6. Ethical Considerations

This study was approved by the Institutional Review Board of CHA University of Medicine and Science (IRB No. 1044308-202412-HR-218-02, 17 February 2025). All procedures were conducted in accordance with the Declaration of Helsinki, and the participant provided voluntary written informed consent after receiving a full explanation of study participation.

## 3. Results

### 3.1. Changes in Isometric Hip Muscle Strength

Marked increases in hip extension strength were observed following the intervention (Table 1). Hip extension peak force increased from 66 N to 235 N on the left (+256%) and from 65 N to 226 N on the right (+247%).

Hip extension RFD also increased substantially on both sides: from 145 N·s^−1^ to 398 N·s^−1^ on the left (+174%) and from 151 N·s^−1^ to 435 N·s^−1^ on the right (+188%).

**Figure 6:**
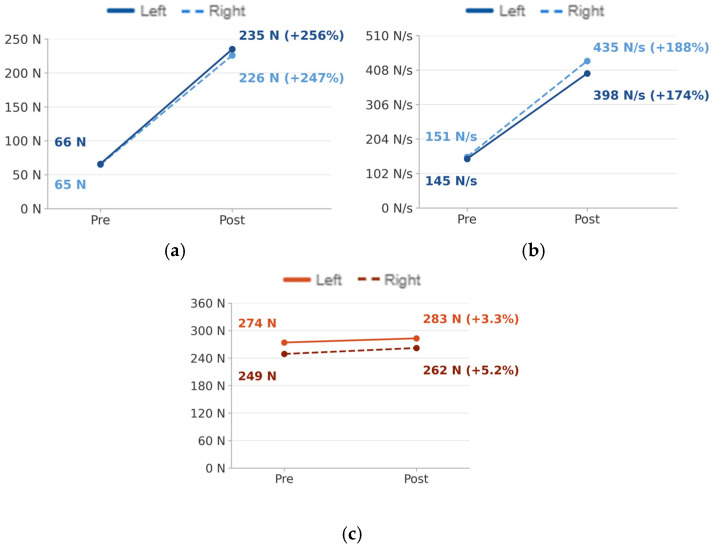
Hip neuromuscular strength (DynaMo) (**a**) Hip extension: peak force (**b**) Hip extension: RFD (**c**) Hip flexion: peak force strength.

In contrast, hip flexion peak force showed comparatively minimal change: a 5.2% increase on the left (249 N to 262 N) and a 3.3% increase on the right (274 N to 283 N). Notably, pre-intervention hip flexion values (249–274 N) substantially exceeded the hip extension values (65–66 N). Although hip extensors are biomechanically stronger in neurologically intact individuals, this pattern is consistent with the upper motor neuron lesion profile observed in HIE, wherein disruption of descending inhibitory pathways leads to flexor hypertonicity and spastic flexor dominance in the lower extremities [21]. This flexion-dominant pattern has been well documented in patients with diffuse hypoxic brain damage who present with quadriparesis, and it should be interpreted within the clinical context of this participant’s neurological profile rather than as a data-entry error. These changes are illustrated in Figure 6.

### 3.2. Changes in STS Performance

**Figure 7:**
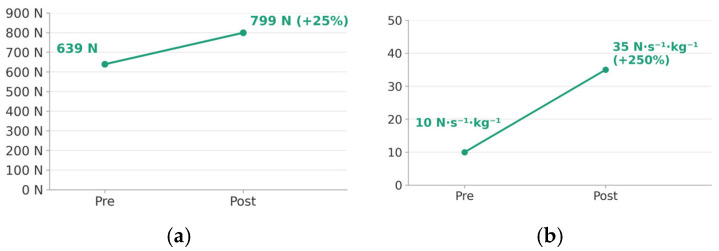
Sit-to-stand performance (ForceDecks) (**a**) STS peak force (**b**) Body mass-normalized RFD.

STS peak force increased from 639 N to 799 N (+25%) following the intervention (Table 2). Body mass-normalized STS RFD increased from 10 N·s^−1^·kg^−1^ to 35 N·s^−1^·kg^−1^ (+250%), representing the largest relative improvement among all outcome variables. These changes are shown in Figure 7.

### 3.3. Frontal-Plane Hip Strength Adaptations

Increases in frontal-plane hip muscle strength were observed following the intervention (Table 3). Hip abduction strength increased by 27.1% on the left and 36.8% on the right. Notably, the inter-limb imbalance for abduction decreased from 14.7% to 6.7%, indicating a trend toward improved bilateral symmetry, with the weaker limb (right) demonstrating proportionally greater gains. Hip adduction strength increased by 43.7% on the left and 14.6% on the right. The inter-limb imbalance for adduction reversed direction—from right dominance (−14.7%) pre-intervention to left dominance (+6.7%) post-intervention—suggesting a substantial shift in frontal-plane neuromuscular activation patterns following the exoskeleton training program.

### 3.4. Changes in Static Balance

**Figure 8:**
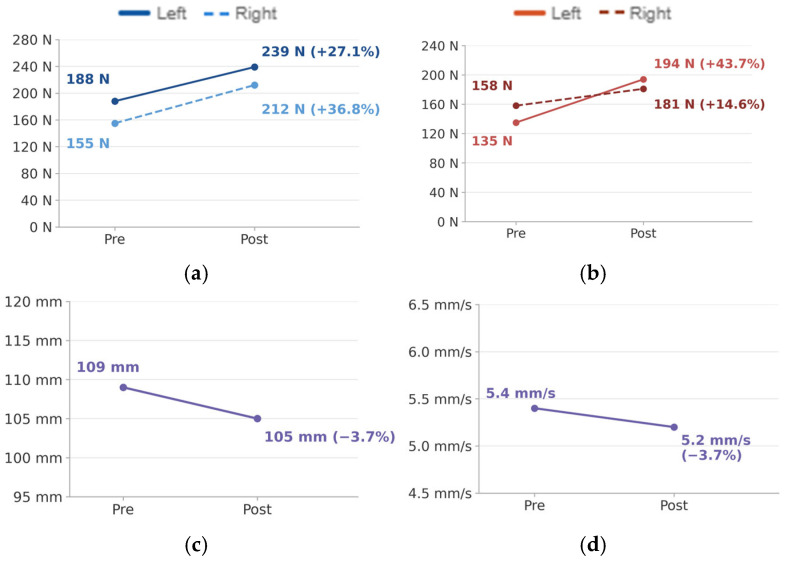
Frontal-plane hip strength & postural stability (**a**) Hip abduction (**b**) Hip adduction (**c**) CoP path length (**d**) CoP mean velocity.

CoP total path length during quiet standing decreased by 3.7% following the intervention (Supplementary Table S1). CoP mean velocity also decreased by 3.7%. No substantial changes were observed in other postural stability indices. The frontal-plane hip strength and postural stability findings are summarized in Figure 8.

## 4. Discussion

### 4.1. Key Findings

This study examined the effects of a six-week powered lower-limb exoskeleton gait training program on hip-level neuromuscular output characteristics and functional performance capacity in a patient with chronic hypoxic-ischemic brain injury using a case report design with pre- and post-intervention evaluation. Bilateral hip extension peak force and rate of force development (RFD) both increased following the intervention, and body mass-normalized RFD during sit-to-stand increased by approximately 250%, indicating a marked improvement in the capacity for rapid initial force generation. These gains were accompanied by concomitant increases in frontal-plane hip muscle strength alongside a reduction in inter-limb imbalance, suggesting both enhanced lateral stability capacity and a trend toward adaptive changes in frontal-plane neuromuscular activation.

These findings indicate that exoskeleton-based gait training may alter hip-centered neuromuscular output strategies beyond simple repetitive ambulation practice.

### 4.2. Neuromuscular Interpretation

The observed increases in RFD may reflect enhanced early motor unit recruitment and improved motor unit firing synchronization, although direct neurophysiological confirmation was not obtained in this study. RFD is a more sensitive indicator of the nervous system’s capacity for rapid activation than maximal muscle strength [5], and may be disproportionately impaired relative to peak force following CNS injury [7,8]. The selective improvement in hip extension RFD observed here is consistent with the task demands of the exoskeleton training paradigm, which repeatedly required hip extension drive during the propulsive phase of gait, plausibly promoting task-specific activation of hip extensor musculature during weight acceptance and propulsion and contributing to adaptive CNS motor control changes. This interpretation is supported by prior work: even two sessions of impairment-specific hip exoskeleton training improved walking distance and speed in patients with acquired brain injury, attributed to enhanced hip extensor engagement during stance [22]; robot-assisted hemiplegic gait rehabilitation has similarly been associated with increased lower-limb muscle strength [23]; and a multicenter RCT in 151 patients with subacute stroke found that overground wearable exoskeleton training produced ambulation gains equivalent to conventional therapy alongside additional lower-extremity strength improvements [24].

The substantial increase in body mass-normalized STS RFD (+250%) suggests more efficient neuromuscular output during functional task performance, consistent with reports that early RFD (0–200 ms) is inversely associated with STS time in chronic kidney disease [25] and that RFD is more sensitive than maximal force for detecting motor impairment severity in multiple sclerosis [26]. The disconnect between minimal gains in hip flexion peak force and substantial gains in hip extension and STS RFD further supports time-dependent force capacity as a distinct, clinically meaningful neuromuscular dimension in this patient with central neurological injury. The reduction in frontal-plane inter-limb imbalance, including a directional reversal of adduction dominance from right to left, is consistent with adaptive changes in the asymmetric neuromuscular activation patterns characteristic of CNS injury [3,4], and aligns with reports that exoskeleton training can promote weight-bearing symmetry and altered muscle activation patterns [18,19].

### 4.3. Clinical Implications

The results of this study suggest that hip-centered neuromuscular output capacity plays an important role in the recovery of functional movement in patients with chronic neurological injury. In particular, RFD is an index closely associated with functional tasks that require rapid force generation within a brief time window—such as sit-to-stand, gait initiation, and weight transfer. Exoskeleton-based gait training therefore represents a potential rehabilitation strategy for improving functional performance capacity through the enhancement of hip-centered neuromuscular output, beyond what simple gait practice alone can achieve. These findings further underscore the need to incorporate time-dependent force metrics such as RFD, alongside maximal muscle strength, into rehabilitation assessments.

### 4.4. Study Limitations

This study was conducted as a case report with pre- and post-intervention evaluation in a single patient, which limits the generalizability of the findings. Because only a single baseline assessment was performed rather than multiple sequential baseline measurements, this design does not meet the criteria for an experimental single-case (single-subject) design; consequently, the possibility that the participant experienced an atypical “off-day” or a period of elevated fatigue specifically during the baseline assessment cannot be ruled out, and the markedly large percentage changes observed (e.g., up to +250%) should be interpreted with this caveat in mind. In addition, the absence of surface electromyography (sEMG) for direct assessment of muscle activation patterns and neurophysiological indices for evaluating corticomotor excitability precluded direct elucidation of the neural mechanisms underlying the observed changes. Spasticity was not assessed before or after the intervention using standardized scales such as the Modified Ashworth Scale; therefore, the possibility that changes in muscle tone—particularly flexor hypertonicity—contributed to the observed gains in isometric force and RFD cannot be excluded, and future studies should incorporate spasticity monitoring to better isolate true strength adaptations. Critically, the DynaMo dynamometer was operated using a hand-held, manual counter-force method by the examiner rather than a mechanically fixed setup. Because accurate quantification of RFD within the early 0–200 ms window requires strict mechanical stabilization, manual counter-force application is highly susceptible to examiner-related variability in resistance onset and stability during this brief time window. The absolute DynaMo-derived RFD values reported in Table 1 should therefore be interpreted with extreme caution, as they may not purely reflect the participant’s neuromuscular capacity and could be confounded by inter-trial variation in manual counter-force application. Mechanical fixation of the dynamometer in future studies is essential to improve the precision and reproducibility of RFD and other time-dependent force time metrics. Furthermore, although a dual force plate system (ForceDecks) was used for STS assessment with each foot placed on a separate plate, individual limb loading data were not extractable from the software output available during the study period; consequently, changes in weight-bearing asymmetry during STS could not be directly quantified, representing a limitation in the interpretation of bilateral symmetry recovery. Additionally, the present study did not include standardized clinical outcome measures of functional independence, gait performance, or fall risk—such as the Timed Up and Go test, 10-Meter Walk Test, or Functional Independence Measure—which would have allowed for direct assessment of whether the observed neuromuscular gains translated into meaningful improvements in activities of daily living; their inclusion in future studies is recommended to establish the clinical relevance of RFD gains in this population. Finally, the majority of references cited reflect the limited availability of contemporary literature on exoskeleton-based rehabilitation in chronic HIE, itself a limitation of the current evidence base. Future research should include prospective controlled studies with larger samples that integrate functional outcome measures, muscle activation patterns, mechanically fixed dynamometry, individual limb loading during STS, gait biomechanics, and neurophysiological indices.

Finally, this report reflects a single six-week training block followed by a single post-intervention assessment, with no extended follow-up. It therefore remains unknown whether the observed gains in RFD and frontal-plane hip strength are stable over time or represent a durable training effect rather than a transient, task-specific adaptation. Longer observation periods with repeated post-intervention assessments are needed before the reliability and long-term clinical significance of these findings can be established. Nevertheless, chronic HIE-induced quadriparesis remains a rare and underreported population, and pre–post intervention data from even a single short-term training block provide hypothesis-generating evidence that is otherwise scarce in the current literature; the selective and internally consistent pattern observed across RFD, peak force, and frontal-plane measures (Table 1, Table 2 and Table 3) further argues against these gains reflecting measurement noise alone. We have therefore framed this report as preliminary, exploratory evidence intended to motivate—rather than substitute for—future controlled studies incorporating extended follow-up.

## 5. Conclusions

Six weeks of powered wearable exoskeleton gait training produced selective and clinically meaningful improvements in time-dependent neuromuscular output—particularly RFD—that exceeded gains in maximal muscle strength, alongside enhanced functional weight acceptance during STS and improved frontal-plane hip muscle symmetry in a patient with chronic HIE-induced quadriparesis. These findings highlight RFD as a sensitive and functionally relevant rehabilitation target that may be inadequately captured by conventional strength assessment alone. Powered exoskeleton gait training shows promise as a rehabilitation strategy capable of driving neuromuscular adaptations beyond what repetitive gait practice alone can achieve in patients with chronic CNS injury. Future controlled studies with larger samples and neurophysiological measures are needed to confirm these findings and elucidate the underlying mechanisms.

## Figures and Tables

**Table 1:** Changes in isometric hip muscle strength and rate of force development (DynaMo).

Variable	Side	Pre	Post	% Change
Hip extension peak force (N)	L	66	235	+256%
	R	65	226	+247%
Hip extension RFD (N·s^−1^)	L	145	398	+174%
	R	151	435	+188%
Hip flexion peak force (N)	L	249	262	+5.2%
	R	274	283	+3.3%

RFD: rate of force development, L: left, R: right.

**Table 2:** Changes in sit-to-stand (STS) performance (ForceDecks).

Variable	Pre	Post	% Change
STS Peak Force (N)	639	799	+25%
STS Peak RFD/BW (N·s^−1^·kg^−1^)	10	35	+250%

STS: sit-to-stand, RFD: rate of force development, BW: body weight.

**Table 3:** Changes in frontal-plane hip strength (ForceFrame).

Variable	Side	Pre (N)	Post (N)	% Change	Imbalance (%) Pre → Post
Hip Abduction Max (N)	L	188	239	+27.1%	14.7% → 6.7% (L dominant; improved)
	R	155	212	+36.8%	
Hip Adduction Max (N)	L	135	194	+43.7%	−14.7% → +6.7% (R → L dominant; reversed)
	R	158	181	+14.6%	

## Data Availability

The data presented in this study are available on request from the corresponding author due to privacy restrictions related to participant data.

## Supplementary Materials

The following supporting information can be downloaded at: https://www.mdpi.com/article/10.3390/brainsci16070688/s1, Table S1: Quiet standing postural stability (ForceDecks).
