# Supplementary material for: Powered Exoskeleton Gait Training and Hip Rate of Force Development in Chronic Hypoxic-Ischemic Encephalopathy: A Case Study
Source: Brain Sci. 2026 Jun 30;16(7):688. doi: 10.3390/brainsci16070688 (PMC13406430; doi:10.3390/brainsci16070688)
Supplement: Supplementary file 1 [file brainsci-16-00688-s001.zip › brainsci-4369903-supplementary.pdf]

Table S1. Quiet standing postural stability (ForceDecks).

Variable	Pre	Post	% Change
CoP Path Length (mm)	109	105	−3.7%
CoP Mean Velocity (mm/s)	5.4	5.2	−3.7%

CoP: center of pressure.
